# The response to PAK1 inhibitor IPA3 distinguishes between cancer cells with mutations in BRAF and Ras oncogenes

**DOI:** 10.18632/oncotarget.587

**Published:** 2012-07-31

**Authors:** Ruchi Singhal, Eugene S. Kandel

**Affiliations:** ^1^ Roswell Park Cancer Institute, Department of Cell Stress Biology, Elm & Carlton St., Buffalo, NY, 142263, USA

**Keywords:** IPA3, PAK1, BRAF, NRAS, KRAS, melanoma, colon cancer, GDC-0879, AZD6244

## Abstract

While new drugs aimed at BRAF-mutated cancers are entering clinical practice, cells and tumors with activating Ras mutations are relatively resistant to those and quite a few other anti-cancer agents. This inspires the effort to reverse this resistance or to uncover new vulnerabilities in such resistant cancers. IPA3 has been originally identified as a small molecule inhibitor of p21-activated protein kinase 1 (PAK1), a candidate therapeutic target in human malignancies. We have tested a battery of melanoma and colon carcinoma cell lines that carry mutations in BRAF, NRAS and KRAS genes and have observed that those with NRAS and KRAS mutations are more sensitive to killing by IPA3. Genetic manipulations suggest that the differential response depends not just on these oncogenes, but also on additional events that were co-selected during tumor evolution. Furthermore, sublethal doses of IPA3 or ectopic expression of dominant-negative PAK1 sensitized Ras-mutated cells to GDC-0897 and AZD6244, which otherwise have reduced efficiency against cells with activated Ras. Dominant-negative PAK1 also reduced the growth of NRAS-mutated cells in confluent cultures, but, unlike IPA3, caused no significant toxicity. Although it remains to be proven that all the effects of IPA3 are exclusively due to inhibition of PAK1, our findings point to the existence of selective vulnerabilities, which are associated with Ras mutations and could be useful for better understanding and treatment of a large subset of tumors.

## INTRODUCTION

Discovery of unique vulnerabilities in cancer cells has emerged as a major starting point for anti-cancer drug development. Serendipitous discovery of cancer-specific toxins is now supplemented, if not yet supplanted, by targeted development of small molecule modulators of specific biochemical functions. Synergistic advances in cancer genetics and in general knowledge of cellular metabolism and signal transduction produce the ever-growing list of desirable therapeutic targets. The corresponding small molecule inhibitors, either empirically discovered or rationally designed, are currently transiting from pre-clinical pipelines into the armamentarium of clinical oncology.

A prominent example of this phenomenon is the emergence of candidate drugs that target various components of MAP kinase cascade. BRAF gene product is targeted by several of such compounds [1, 2] and is abnormally activated in various cancers, including approximately half of all human melanomas [3-5] (discussed in [6]). Selective reliance on BRAF activity by cancer, but not the normal cells, makes BRAF inhibitors relatively safe and efficient against a subset of melanomas, which are hardly amenable to conventional chemotherapy[7]. Unfortunately, a significant subset of melanomas and the majority of other tumors are initially BRAF-independent, while BRAF-mutated cancers eventually develop resistance in the course of therapy [7]. For example, resistance may arise from mutations in Ras oncogenes [8] which, apparently, supply a signal that is equivalent to, but is independent of, BRAF mutations. This sustains the interest to discover BRAF-independent vulnerabilities in these malignancies.

P21-activated kinases or PAKs are a family of evolutionary conserved enzymes that were originally identified as downstream effectors of Rho GTPases. Group I PAKs, which include PAK1, PAK2 and PAK3, have the same general architecture, but distinct and, occasionally, opposite biological roles. PAK1 in particular has been associated with a variety of pathological conditions, including cancer (reviewed in [9]). PAK1 is an intermediate in several pathways, whose perturbation is known to be oncogenic. For example, it is known to be downstream of Rac1 [10], which, in turn, is activated by oncogenic Ras proteins. PAK1 function has been directly implicated in several aspects of Ras-mediated transformation, at least in some rodent cell lines [11, 12]. PAK1 is also affected by PI3K pathway [13-18] and, possibly, directly interacts with protooncogene Akt [15, 18]. Among PAK1 targets are Raf proteins [19, 20], which may explain PAK1 connection to MAPK cascade. Finally, PAK1 has been implicated in several anti-apoptotic mechanisms, as well as in the control of various metabolic processes (reviewed in [9]). Overexpression or activation of PAK1 has been reported in a large number of malignancies and, not surprisingly, this kinase is recognized a potential target for cancer therapy. A notable step in that direction was a search for the inhibitors of the interaction of PAK1 with its activator molecules [21]. Although the main discovery of this study, a compound designated IPA3, does not have chemical properties suitable for clinical use, it became an affordable and convenient tool to manipulate PAK1 activity in cell culture models (e.g. [22-26]), because it allows one to quickly probe the dependence of various phenomena on PAK1. In the current study, we have examined the differential effects of IPA3, either alone or in combination with some other MAPK cascade inhibitors, on cancer lines with known mutations in Ras and BRAF genes.

## RESULTS

Genetic data suggests that mutations in Ras and BRAF oncogenes are somewhat equivalent during the early stages of tumor development. This is inferred from, generally, mutually exclusive occurrence of these changes [27, 28]. NRAS is the most commonly mutated Ras family member in melanoma, KRAS is predominantly mutated in colon cancer, while BRAF mutations are found in either malignancy. While either of these mutations may lead to hyperactivation of the MAPK cascade, they are likely to differ in the spectrum of additional changes inflicted on the affected cell. In turn, this may result in selective pressure to acquire different secondary mutations and on differential dependence of various otherwise normal cellular factors. Thus, each of these mutations may create its unique pattern of vulnerabilities and resistances to potentially cytotoxic impacts.

**Figure 1:**
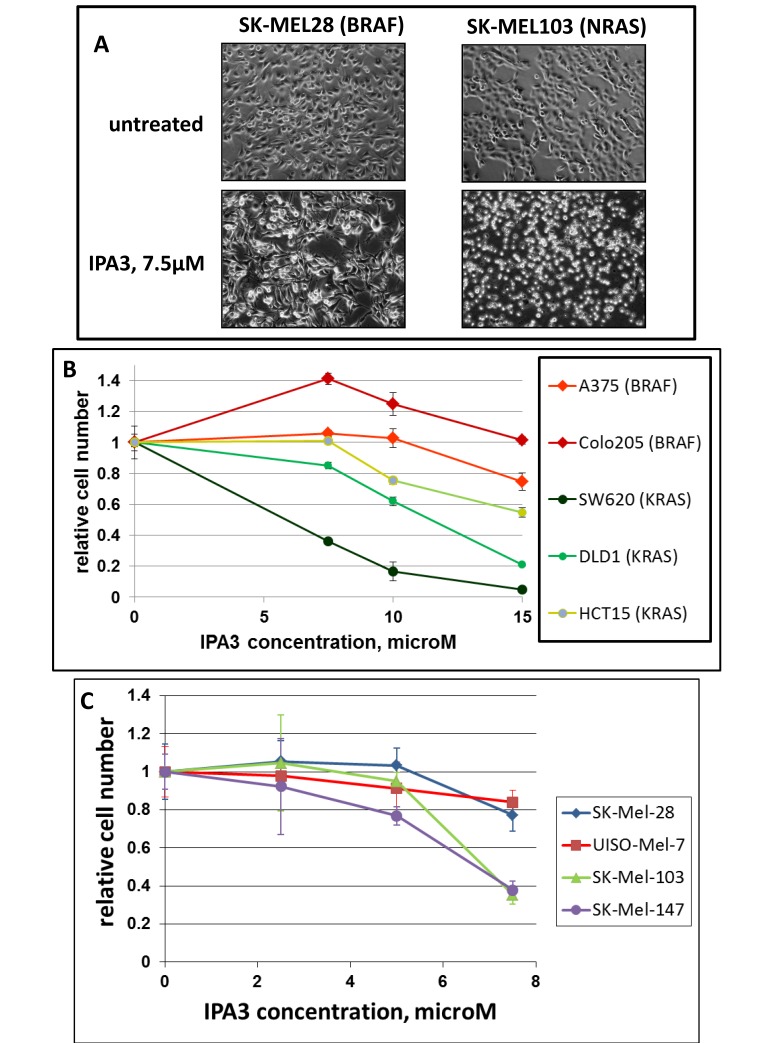
Differential sensitivity of BRAF- and RAS-mutated cells to IPA3 Indicated cell lines (mutated oncogenes are shown in parentheses) were plated at the same density (30000 cells/well) in 12-well plates and next day treated with various concentrations of IPA3. Two days later, images of the remaining cells were taken (A), and the plates were fixed and the cells were quantified (B and C) by methylene blue staining/extraction method as described in Methods. The number of remaining viable cells is displayed relative to the number of cells in parallel untreated cultures of the same cell line. Each data point was collected in triplicates, and the standard deviations are denoted by error-bars.

We examined five melanoma lines, three of which were BRAF- and two NRAS-mutated, and four colon carcinoma lines, of which three were KRAS- and one BRAF-mutated. The cells were treated with various doses of IPA3 and the response was recorded by microphotography and by methylene blue staining and extraction method (Figure 1A, B and C). We observed that Ras-mutated cells, either melanomas or colon carcinomas, were killed by the doses of IPA3, which affected the morphology, but were hardly the viability of BRAF-mutated cells of similar origin. In fact, we have repeatedly seen that low doses of IPA3 had a small stimulating effect on proliferation of some BRAF-mutated cell lines (Figure 1B and C, and unpublished). Overall, all BRAF-mutated lines were more resistant than any of the Ras-mutated ones, indicating a significant (p<0.02 by Mann-Whitney test) difference between these two groups of cell lines.

**Figure 2:**
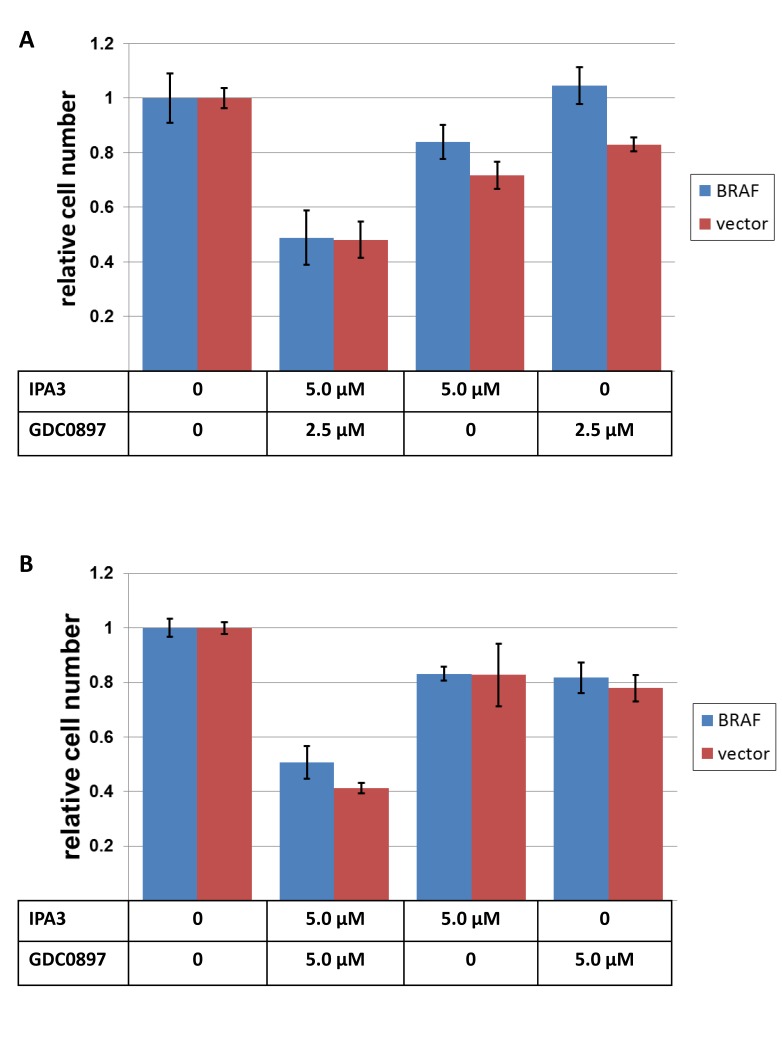
The effects of IPA3 and GDC-0897 on NRAS-mutated melanoma cell lines SK-MEL-103 (A) and SK-MEL-147 (B), transduced either with a BRAF-V600E – expressing construct (“BRAF”) or with the corresponding empty vector (“vector”), were plated at 30000 cells/well in 12-well plates and treated next day with the indicated doses of IPA3 and GDC-0897. 72 hours later, the remaining viable cells were fixed and quantified as in Figure 1.

**Figure 3:**
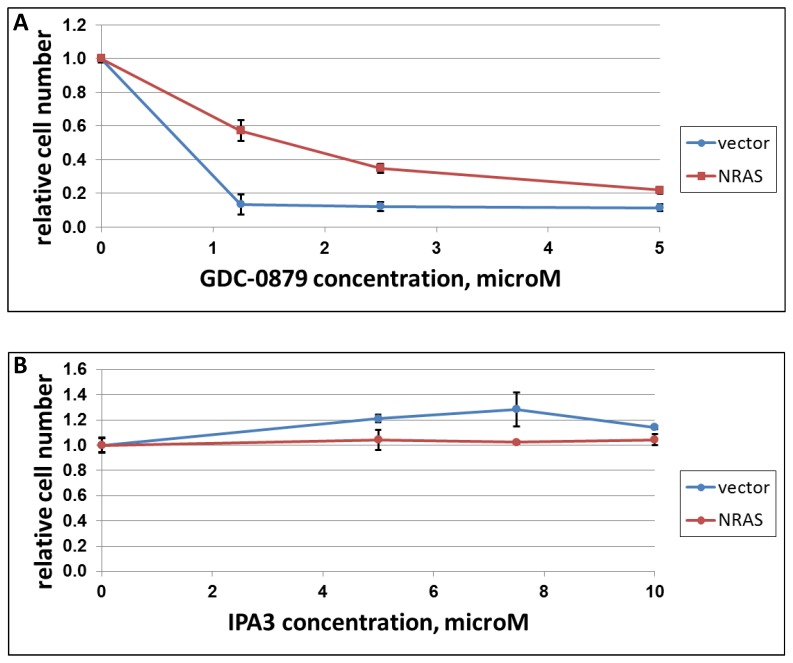
The effect of NRAS on response of A375 cells to IPA3 and GDC-0897 Melanoma cell lines A375 (BRAF-mutated) was transduced with a construct expressing activated NRAS (“NRAS”) or the respective vector control (“vector”). The cells were plated in 12-well plates (30000/cells per well) and treated next day with indicated doses of GDC-0897 (A) or IPA3 (B). Three days later, the remaining viable cells were quantified as in Figure 1.

The connection between oncogenic Ras and such downstream effectors as ERKs is believed to be principally mediated by CRAF kinase, which, in turn, supports the activity of ERK activators, MEKs. However, other Raf kinases, such as BRAF, could still be present in the same cell. Although some functions of CRAF and BRAF are similar, the contribution of BRAF to growth and survival of such cells is expected to be overshadowed by that of Ras/CRAF axis. This is best attested by insensitivity of such cells to BRAF inhibitors. We have examined whether IPA3 treatment affects the response of Ras-mutated lines to BRAF inhibitor GDC-0879[29]. As expected, two NRAS-mutated melanomas showed little response even to relatively high doses of the drug (Figure 2A and B). In fact, the doses of 2.5μM and 5μM of GDC-0879 are much higher that the IC50 for BRAF-mutated lines ([29] and Figure 3A). Remarkably, in the presence of otherwise sublethal doses of IPA3, addition of the BRAF inhibitor to otherwise resistant cells had a pronounced suppressive effect, indicating that the two compounds cooperate.

In a reciprocal experiment, ectopic expression of activated NRAS protected cells from BRAF inhibitor GDC-0879 (Figure 3A), but failed to make them sensitive to IPA3 (Figure 3B). We concluded that the distinct response to the latter compound is, at least in part, dictated by co-selected mutations rather than by different direct effects of BRAF and Ras oncogenes.

**Figure 4:**
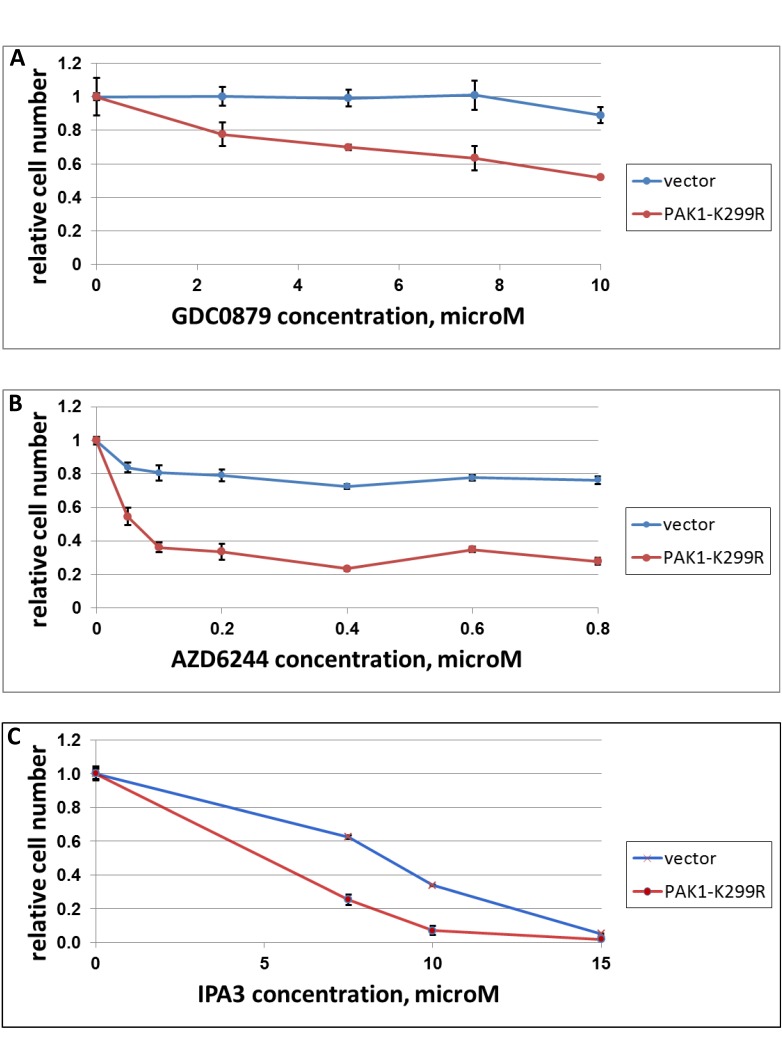
The effect of dominant-negative PAK1 on response of A375 cells to GDC-0897, AZD6244 and IPA3 Melanoma cell lines SK-MEL-103 (NRAS-mutated) was transduced with a construct expressing dominant-negative PAK1 (“PAK1-K299R”) or the respective vector control (“vector”). The cells were plated in 24-well plates (30000/cells per well) and treated with indicated doses of GDC-0897 (A), AZD6244 (B) or IPA3 (C). Three days later, the remaining viable cells were quantified as in Figure 1.

**Figure 5:**
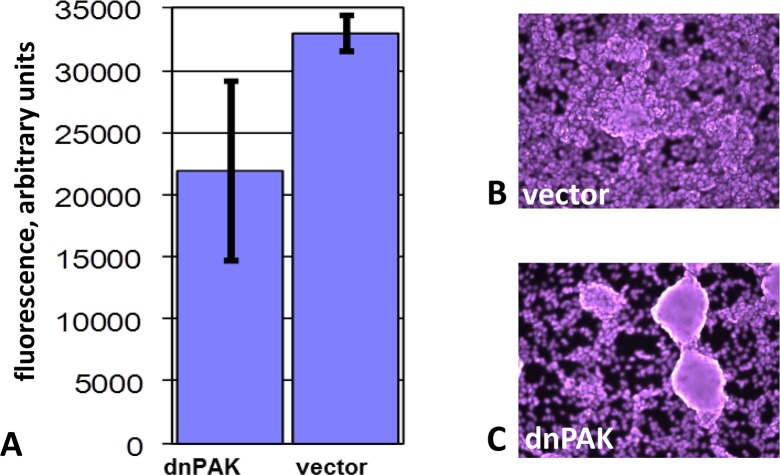
The effect of dominant-negative PAK1 on growth of SK-MEL-103 cells in confluent cultures A. SK-MEL-103 cells transduced with a construct expressing dominant-negative PAK1-K299R (“dnPAK”) or the respective vector control (“vector”) were cultured for two days after reaching confluence. Three wells of each variant were fixed and stained with Hoechst 33342, and readings were taken from 25 points on each of the wells. The average value (in arbitrary units) of all the readings for each cell line is shown with standard deviation. B-C. The indicated cell lines were cultured for six more days after reaching confluence, fixed and stained with Hoechst 33342 and photographed under a fluorescent microscope. Note the change from uniform multilayer growth to individual foci.

We used a dominant-negative form of PAK1 (PAK1-K299R) as an alternative way of affecting the function of PAK1 in Ras-mutated cells. In comparison with an empty vector, PAK1-K299R sensitized cells to GDC-0879 (Figure 4A), AZD6244 (an inhibitor of MEK 1 and 2) (Figure 4B) and IPA3 (Figure 4C). In contrast to IPA3, PAK1-K299K did not exhibit a noticeable cytotoxic or cytostatic effect under normal culture conditions. However, it reduced the growth of SK-MEL-103 (NRAS mutant) in confluent cultures (Figure 5A-C). While the control cells after reaching confluence continued uniform growth as a multilayer culture (Figure 5B), a large fraction of cells transduced with PAK1 mutant appeared contact-inhibited and only a lesser fraction continued proliferation and formed foci (Figure 5C). Of note, we have previously observed the ability of PAK1-K299K to reduce transformed phenotype, but not the viability, of cells transduced with an oncogenic variant of Akt [13].

## DISCUSSION

Differential sensitivity to IPA3 in Ras-mutated cell lines and the ability of this compound, even in sublethal concentrations, to sensitize these cells to other impacts point to an important vulnerability, which might be clinically explored in a single-agent or combined therapy. The biochemical basis of the difference in drug response between BRAF- and Ras-mutated cells remains unclear. Our observations suggest that this phenomenon is not a direct consequence of the activation of these oncogenes: when we attempted to transfer the oncogenes between the cell lines, the status of IPA3 resistance did not follow the transgene. It is tempting to speculate, that a PAK1-like function is critical to these cancers, but in Ras-mutated cells the signal to PAK1 originates directly from Ras, while BRAF needs a cooperating event to achieve the same goal. Assuming that the cooperating event is IPA3 insensitive, this hypothesis agrees with our observations, although alternative explanations are possible as well. The scenario, in which genetic events that provide alternative solutions to the same hurdle in the early cancer development steer the evolution of the disease into significantly different paths, is well-known from other systems (e.g. [30])

An important issue in this and other works that utilize IPA3 is how specific this compound is. Its original characterization included a diligent study of more than two hundred other kinases, of which only a handful were affected [21]. However, the few that were inhibited, along with PAK2 and PAK3, which are also susceptible to IPA3, have credible connections to cancer and cell death (discussed in [9]). In this regard, the experiments with alternative modes of PAK1 inhibition become important. A recent paper reported concordant toxicity of IPA3 and an anti-PAK1 shRNA in a number of cell lines [26], arguing in favor of IPA3 specificity.

In our experiments, dominant-negative PAK1 resembled IPA3 in that it was able to sensitize Ras-mutated cells to a BRAF inhibitor. Although GDC-0897 is very specific against BRAF-mutated cells [29], we cannot rule out that the observed effect is due to a residual activity of GDC-0897 against CRAF. The issue of BRAF inhibitors having some potency against CRAF is well recognized for clinically used compounds[2], and the observed activity of IPA3 may be significant if it proceeds either through BRAF or CRAF inhibition.

The same transgene also sensitized to IPA3 itself, which would be expected if the two impacts target the same biochemical process. Sensitization to AZD6244 (aka Selumetinib) is in particular important, as it points to an avenue of increasing the efficacy of the compound, which is currently entering clinical practice. This may be especially interesting in the view of the earlier reports that Ras-mutated cells are, generally, more resistant to MEK inhibitors than the BRAF-mutated ones [31, 32], and this trend is seen upon clinical use of AZD6244[33]. The effect of PAK1-K299R on the response to the inhibitors of MAPK cascade is not surprising, since PAK1 has been implicated in regulation of and in direct interaction with various components of this pathway[13, 19, 20, 34, 35]. In addition, PAK1 has been implicated in various modes of protection from apoptosis [36-40], and a reduction in such an activity may have contributed to the observed sensitization phenomena, although at the doses and treatment times used in our experiments AZD6244 and GDC-0897 are predominantly cytostatic (data not shown).

It is important to note that expression of PAK1-K299R did not recapitulate the pronounced toxicity of IPA3. The most trivial explanation for our results is that the expression of the transgene varied between individual infected cells and, on average, was insufficient to achieve complete inhibition of the endogenous PAK1. This may explain why confluent cultures of those cells appeared more heterogeneous according to the measurements of cell density (Figure 5A) and visual appearance (Figure 5C). Another possibility is a difference in the ability of IPA3 and PAK1-K299R to suppress different Group I PAKs or the proposed kinase-independent functions of PAK1 [41]. Nevertheless, it is impossible to completely rule out that the difference between the effects of the dominant-negative PAK1 and IPA3 is, at least in part, due to some unknown PAK1-independent activity of the latter.

An unbiased genetic screen [42] for the events that protect cells from the toxicity of IPA3 may be warranted in order to uncover critical molecular events, either PAK1 dependent or not, that are triggered by this compound in cancer cells. Identification of such events would be an important aid in developing new and, hopefully, clinically-applicable compounds with an IPA3-like effect on cancer cells. As PAK1 itself has clearly emerged as a target for cancer therapy (discussed in [9, 43]), the knowledge of the ways, in which otherwise sensitive cells may gain tolerance to inhibitors of this kinase, is likely to rise in clinical significance.

## METHODS

The cancer cell lines used in this study included: BRAF-mutated melanomas (UISO-MEL-7, SK-MEL-28, A375), NRAS-mutated melanomas (SK-MEL-130, SK-MEL-147), BRAF-mutated colon carcinoma (COLO-205), KRAS-mutated colon carcinomas (DLD-1, HCT-15). All cells were cultured in humidified chambers at 37°C and 5% CO2 in high-glucose DMEM supplemented with L-glutamine (4 mM), fetal bovine serum (10%), penicillin (100 U/ml) and streptomycin (100 μg/ml). Cells were free of mycoplasma contamination, as tested using MycoAlert Mycoplasma Detection Kit (Lonza).

Recombinant retroviral stocks were produced by co-transfecting 293T cells with an appropriate vector and a packaging construct (pCL10A1 from Imgenex, Inc). Transfections were carried out using Lipofectamine Plus (Life Technologies Corporation) according to the manufacturer's recommendations. Viral stocks were applied as previously described [44].

Dominant-negative PAK1 expression vector was described earlier [13]. Constructs for the expression of activated human NRAS and BRAF were gifts of Drs. M. Nikiforov and J. Kichina, respectively (both of Roswell Park Cancer Institute).

Relative cell numbers were compared using methylene blue staining/extraction method. Briefly, cells were rinsed with phosphate-buffered saline, fixed in methanol and stained with a 2% solution of methylene blue in a 1:1 mix of water and methanol. Unincorporated dye was washed away with deionized water; the remaining dye was extracted in 1.0 M HCl and quantified by spectrometry at 600 nM.
